# Economic and budgetary impact evaluation of isavuconazole (Cresemba^®^) versus voriconazole (Vfend^®^) for the treatment of patients with possible invasive aspergillosis from the perspective of the Brazilian supplementary health system

**DOI:** 10.1371/journal.pone.0299056

**Published:** 2024-03-01

**Authors:** Gisele Lemes Veiga Araujo, Laura Murta Amaral, Vinicius Ponzio, Jaime Luis Rocha

**Affiliations:** 1 Knight Therapeutics, São Paulo, Brazil; 2 ORIGIN Health, São Paulo, Brazil; 3 Federal University of São Paulo, UNIFESP, São Paulo, Brazil; 4 Faculty of Medicine of the Pontifical Catholic University of Paraná, PUC, Paraná, Brazil; Gulu University, UGANDA

## Abstract

**Objectives:**

This study aims to evaluate the cost-utility and the budgetary impact of isavuconazole compared to voriconazole in patients with suspected invasive aspergillosis (IA) from the perspective of the Brazilian supplementary health system (SHS).

**Methods:**

In this model, a decision tree was developed and included patients with possible IA. Efficacy parameters were extracted from the clinical studies. Drug acquisition, hospitalization costs and adverse events were also collected. Alternative 3- and 10-year time horizon scenarios were used. In addition, deterministic and probabilistic sensitivity analyses were simulated. A budget impact analysis of isavuconazole versus voriconazole was performed, assuming a time horizon of 5 years. In addition, sensitivity analyses were conducted to assess the robustness of the model. Results are reported in Brazilian Real (BRL), year values 2022.

**Results:**

The economic analysis of the base case showed that isavuconazole is associated with a saving of 95,174.00 BRL per patient compared to voriconazole. All other simulated scenarios showed that isavuconazole is dominant versus comparators when considering a willingness to pay 40,688.00 BRL/Quality-Adjusted Life Years (QALY). The results were considered robust by the sensitivity analyses. The budget impact analysis showed that the incorporation of isavuconazole generates savings to the SHS, compared to voriconazole, of approximately 20.5 million BRL in the first year. This reaches about 54 million BRL in the fifth incorporation year, considering the market penetration of 20% in the first year, and 50% in the fifth year.

**Conclusion:**

Compared with voriconazole, isavuconazole is regarded as a dominant treatment strategy for patients with suspected IA and generates savings for the SHS.

## Introduction

Systemic triazoles within the azole class are widely used antifungals due to their superior safety profile compared to polyenes [1]. One of the newest triazoles is isavuconazole, approved for the treatment of invasive aspergillosis (IA) and mucormycosis (IM) by the U.S. Food and Drug Administration (FDA) in 2015 and by Brazilian Health Regulatory Agency (ANVISA) in 2019.

Invasive fungal infections (IFIs) are associated with a substantial impact on different populations. A study in 8 reference centers concluded that Brazil has a high incidence and mortality rate due to IFI in patients with acute myeloid leukemia (AML) or myelodysplasia who received chemotherapy and hematopoietic cell transplant (HCT) recipients. The main infections in these patient groups were fusariosis and IA [2].

Another retrospective cohort study conducted in a tertiary hospital in the city of São Paulo (SP), Brazil, demonstrated 94 cases of IFI among 664 hematological patients and 316 HCT recipients. The frequency among patients undergoing allogeneic hematopoietic stem cell transplantation or autologous hematopoietic stem cell transplantation diagnosed with acute leukemia or other hematological malignancies was 8.9%, 1.6%, 17.3%, and 6.4%, respectively. IA was the IFI with the highest incidence (53.2%), followed by fusariosis (18.1%), candidiasis (10.6%), and cryptococcosis (8.5%). Other etiologies for lower incidence cases were hyalohyphomycosis (4.2%), IM (2.1%), *Penicillium sp*. (2.1%), and trichosporonosis (1%) [3].

Isavuconazole has many advantages over other antifungal drugs, including intravenous (IV) and oral formulation, broad-spectrum activity, predictable pharmacokinetics, and reduced adverse effects compared to other triazoles. Isavuconazole is an excellent alternative to voriconazole for IA in patients with hematological malignancies with significant concerns for drug interactions and toxicities [4], as demonstrated in the SECURE clinical trial.

SECURE was a phase 3, double-blind, global multicentre, comparative-group study where patients with suspected invasive mould disease were randomised in a 1:1 ratio, stratified by geographical region, allogeneic haemopoietic stem cell transplantation, and active malignant disease at baseline, to receive isavuconazole or voriconazole. A total of 527 adult patients were randomly assigned (258 received study medication per group) between March 7, 2007, and March 28, 2013. All-cause mortality from first dose of study drug to day 42 for the ITT population was 19% with isavuconazole (48 patients) and 20% with voriconazole (52 patients), with an adjusted treatment difference of -1.0% (95% CI -7.8 to 5.7). Because the upper bound of the 95% CI (5.7%) did not exceed 10%, non-inferiority was shown [8].

Some economic studies have demonstrated that isavuconazole compared to voriconazole is a cost-effective technology for treating suspected IA [5–7]. However, to date, no economic evaluation has been performed from the perspective of the Brazilian health system. The study aimed to develop an economic and budgetary impact analysis of isavuconazole versus voriconazole in patients with suspected IA from the perspective of the Brazilian supplementary health system (SHS).

## Methods

A cost-utility analysis from the Dental and Pharmaceutical Benefits Agency of Sweden (TLV) was adapted for the Brazilian SHS with data available from the literature. It is noteworthy that the same economic modeling rationale was used in the publications by Floros et al. (2019) in Sweden, Azanza et al. (2021) in Spain, and Beauchemin et al. (2022) in Canada [5–7].

In the analysis a certain proportion of people presumed of having IA, in reality had mucormycosis. However, given the difficulties in achieving prompt differential diagnosis, it was assumed that antifungal treatment was initiated before pathogen information was available to clinicians. For a proportion of these patients, this information was assumed to become available during their treatment course, while for the remainder a differential diagnosis was not achieved.

Considering that previously published information was used, the appreciation by an ethics committee, as well as the need for an informed consent, were waived.

### Population

The population was composed of suspected IA patients, according to the inclusion criteria described in the SECURE study, where patients 18 years or older were eligible if they were considered to have invasive mould disease by meeting the criteria for proven, probable, or possible invasive mould disease caused by *Aspergillus spp* or other filamentous fungi [8]. The patients did not present clinical characteristics that defined the diagnosis as IA or IM.

The data of Bergamasco et al. (2021) study demonstrated that SECURE population represent the Brazilian population [3] and Brazilian infectologists were consulted too.

### Comparator

The comparator used in the economic evaluation was voriconazole (reference), recommended by clinical guidelines ECIL-6 (2017) and ESCMID-ECMM-ERS (2017) [9,10] and used in clinical practice to treat suspected IA patients. It’s important to state that Brazil doesn’t have national guidelines for IFIs.

### Time horizon

The 5-year time horizon in the economic analysis considered the life expectancy of patients with AML, the population predominant in the SECURE study [8]. As other conditions can interfere with a patient’s life expectancy, 3- and 10-year horizons were tested as alternative scenarios. This study over time horizons of 3 and 10 years was conducted with the extrapolation of isavuconazole and voriconazole using efficacy and costs.

### Utility

It was considered that IFI survivors would experience the quality of life and life expectancy associated with their underlying condition. The most common underlying disease for patients in the SECURE trial was AML. Therefore, based on an analysis of disease survivors, the utility value for the base case analysis was 0.82, EQ-5D weight elicited from 88 acute myeloid leukemia survivors of Leunis (2014) study [11]. It’s important to state that Brazil doesn’t have utility data defined.

### Model structure

**Fig 1:**
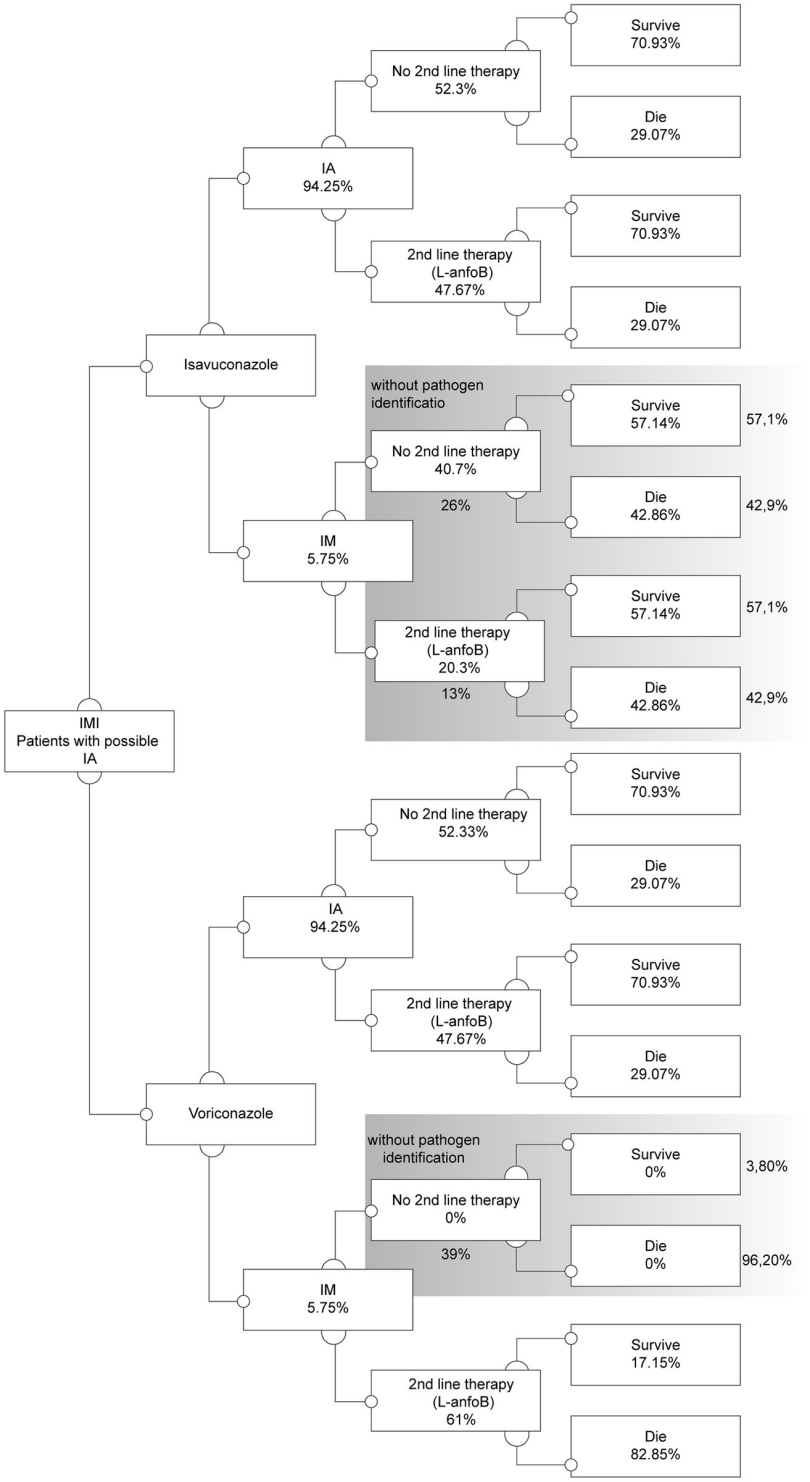
Model design. First level decision nodes represent the treatment comparison, second level decision nodes represent the IA/mucormycosis pathogen split, third level decision nodes are associated with second-line treatment options, areas in grey represent the parts of the tree branch where pathogen information has/may have an effect on treatment decisions. IMI: invasive mold infection; IA: invasive aspergillosis; IM: invasive mucormycosis; L-anfB: liposomal amphotericin B.

A decision tree was developed to evaluate the isavuconazole cost-utility compared to voriconazole in treating patients with suspected IA. The model reflects a population of patients with suspected IA who can be treated with isavuconazole or voriconazole. The disease course simulation considered that suspected IA patients entered the model, assuming that 5.75% of them had IM; this was sourced from a large population-based analysis calculating the 10-year trend of IFIs’ incidence in France [12] and it was tested in univariate sensitivity analysis to reflect the incidence of IM in the Brazilian population in different regions of the country. Then, they can be treated with isavuconazole or voriconazole, after six days the causative pathogen of IFI is identified as *Aspergillus* or *Mucorales*. It was also assumed that information about the pathogen would be available during the treatment course (six days) only to 61% of patients [2,5]. Thus, it was considered that the remaining patients had an unavailable differential diagnosis. The patients can follow the treatment or change for the second-line treatment with liposomal amphotericin B (L-anfB). The change to second-line treatment occurred when patient did not respond to the first-line treatment. However, all confirmed IM patients in the voriconazole arm switch to second-line treatment since voriconazole is not indicated for treating *Mucorales* [13,14]. In the sequence the patient follows to survive or death (Fig 1). Patients with IM who did not change treatment lines failed to obtain a pathogen confirmation and, therefore, remained inadequately treated for a short period (Fig 1 and Table 1).

**Table 1:** Clinical data.

Treatment	Pts referred to 2^nd^ line of treatment (%)	All-cause mortality (%)	Treatment duration (days)	Source
**IA** **Isavuconazole**	47.67	29.07	47	SECURE trial–Total number of days of therapy; based on the SECURE trial clinical study report [8]
**Isavuconazole (1**^**st**^ **line treatment response)**	-	-	77.1	Assumption–Adjustment using the mean treatment duration for the entire cohort and that in those no response to 1^st^ line treatment
**Isavuconazole (no response–prior to switching)**	-	-	14	Assumption [5]
**Voriconazole**	47.67	29.07	47	Assumption–Assumed equivalent to observed value for isavuconazole in SECURE trial. [8]
**Voriconazole (1**^**st**^ **line treatment response)**	-	-	77.1	Assumption–Adjustment using the mean treatment duration for the entire cohort and that in those no response to 1^st^ line treatment
**Voriconazole (no response–prior to switching)**	-	-	14	Assumption [5]
**L-anfB**	-	29.07	47	Assumption–Assumed equivalent to observed value for isavuconazole in SECURE trial. [8]
**L-anfB (treatment response)**	-	-	77.1	Assumption–Assuming treatment duration is equivalent to those responding to isavuconazole
**IM** **Isavuconazole**	33.33	42.86	149	VITAL trial–Total duration of therapy in the primary therapy group; based on VITAL trial [15]
**Isavuconazole (1**^**st**^ **line treatment response)**	-	-	216.5	Assumption–Adjustment using the mean treatment duration for the entire cohort and that in those no response to 1^st^ line treatment
**Isavuconazole (no response–prior to switching)**	-	-	14	Assumption–Assumed to be equivalent to IA
**L-anfB**	-	-	216.5	Assumption–Assumed to be equivalent to isavuconazole
**Voriconazole**	100	-	47 (no pathogen information) and 6 (pathogen information to IM and switching)	Assumption–Used in the scenario when no pathogen information is available. Equivalent to isavuconazole in IA and switching after 6 days when pathogen information is available [5]
**Delayed L-anfB therapy**	-	82.85	-	-
**Untreated**	-	96.20	-	-

IA: invasive aspergillosis; IM: invasive mucormycosis; L-anfB: liposomal amphotericin B.

### Clinical data

#### Second-line treatment

The percentage of patients who received second-line treatment was calculated from the SECURE [8] and VITAL [15] trial data. Patients who did not respond to treatment and had adverse events or disease complications were considered participants who discontinued first-line treatment.

It was assumed that 47.67% of patients switched to second-line treatment due to IA identification [8]. The percentage was used in both model arms, isavuconazole and voriconazole, because there was no statistical difference. However, in the case of IM, the switch rate for the second-line treatment was 33.33% for the isavuconazole arm and 100% for the voriconazole arm (Table 1).

#### Mortality

All-cause mortality for patients identified with IA was assumed as 29.07% for both analysis arms, as there was no statistical difference in the SECURE study, including for the second-line treatment [8].

For patients identified with IM, the mortality rate used was 42.86% from the VITAL study isavuconazole arm [15], 82.85% from the isavuconazole/L-anfB arm due to delayed treatment [16], and 96.20% for patients not adequately treated [17] (Table 1).

### Treatment

#### Dose

The recommended dose in package leaflets was used for each treatment. The mean population weight of the SECURE study [8] of 71.41Kg (± 16.37) was considered to calculate doses, as this information reflects Brazilian population characteristics.

It was considered that 75% of patients started treatment by the IV route for the IA population, according to data from the VITAL study [15], while 25% of patients started treatment orally. In the IM population, 100% of the patients started treatment with L-anfB by IV route. In Brazil, posaconazole in IV and oral presentations (tablets and/or capsules) are not approved, only in the solution pharmaceutical form, indicated for mouthwash and not for the IM treatment.

#### Treatment time

Treatment durations are summarized in Table 1. We considered the isavuconazole treatment duration according to data from the SECURE trial [8] (47.0 days in total: 8.1 IV days, 38.9 days oral) for patients with IA, adjusted according to the cohort mean treatment time considering patients who responded and remained on first-line treatment, and those who discontinued and switched to second-line therapy. Since there are no statistical differences between therapies, we assumed the total treatment duration of 77.1 days for IA for both therapies. The duration of second-line therapy was considered equal to that of first-line therapy in patients who responded to treatment (77.1 days).

The day of change to second-line treatment was assumed to be the 14^th^ treatment day. Thus, an IA patient who started voriconazole treatment and switched to second-line therapy would be on treatment for 14 + 77.1 days. Specifically, the duration of L-anfB treatment was assumed as 14.5 days, and oral voriconazole, 62.6 days, was calculated from the subtraction of 14.5 days from general second-line treatment duration (77.10 days).

The total treatment duration for IM patients was adjusted as for IA, based on data from the VITAL study (IV, 15.5 days; oral, 133.5 days) [15]. Therefore, the total treatment duration assumed for IM patients receiving isavuconazole was 216.5 days.

The same assumptions regarding the duration of second-line IV and reduction therapy were made as for IA. The L-anfB treatment duration was 216.5 days, assumed to be equivalent to the isavuconazole treatment duration.

According to the model, IM voriconazole-treated patients with treatment change after six days due to pathogen confirmation have a total treatment time of 6 days + 216.5 days. In patients without pathogen confirmation, the total treatment duration was assumed as 47 days.

### Cost data

Only direct costs were included in the model, such as drug acquisition, hospitalizations, adverse events (AEs), and laboratory analysis costs. All costs are from 2022 and expressed in Brazilian real (BRL).

#### Drug acquisition

We considered the factory price plus an 18% of Tax on the Circulation of Goods and Services (ICMS) for the drug acquisition. These prices were obtained in the table published by the Chamber of Regulation of the Drug Market (CMED) in May 2022 [18], according to Table 2.

**Table 2:** Doses and cost of drug acquisitions.

Formulation	Unit size	Pack size	Price (BRL)	Dose per day
**Isavuconazole**				
Capsules	100 mg	14 tablets	4.572	Day 1 and 2: 600mg Day 3 onwards: 200 mg
IV	200 mg	1 vial	1.735	Day 1 and 2: 600mg Day 3 onwards: 200 mg
**Voriconazole**				
Tablets	200 mg	14 tablets	6.952	Day 1 and 2: 800mg Day 3 onwards: 400 mg
IV	200 mg	1 vial	1.688	Day 1 and 2: 857mg Day 3 onwards: 571 mg
**L-anfB**				
IV	50 mg	10 vials	24.011	357 mg

BRL: Brazilian real; IV = intravenous. Source: adapted from CMED.

#### Hospitalization

The frequency of hospitalization for IA patients was obtained through the average initial length of stay observed in the SECURE study [8] for isavuconazole (mean, 18.6 days) and voriconazole since the difference between treatments was not statistically significant. For second-line treatment with L-anfB followed by voriconazole, it was assumed that the length of stay would be similar to the average duration for all SECURE trial patients, i.e., 18.6 days [8].

The frequency of hospitalization for IM patients were estimated through the initial average length of stay observed in the VITAL study for isavuconazole (mean, 19.3 days) [15]. The average length of stay was defined as equal to the IV therapy duration for patients treated with L-anfB/voriconazole observed in the FungiScope^™^ case-control study. In this study, patients receiving IV therapy for IM would remain hospitalized until they could change IV administration to an outpatient setting (mean, 27.2 days). Hospitalization during complete treatment before switching (mean, six days) was assumed for patients receiving voriconazole before L-anfB. According to the IM treatment protocol, hospitalization was assumed for patients without information on pathogens and treated with voriconazole.

The cost per day used in the model was 5,520.32 BRL for hospitalization for IA or IM from UNIDAS report [19], referring to a December 2020 daily rate corrected by December 2022 General Market Price Index (IGP-M, FGV) [20].

#### Adverse events

The model included the estimated costs of moderate to severe adverse events (AEs) from the SECURE study [8] that showed significant differences and economic impact. These AEs are from cardiac and hepatobiliary systems classes of organs.

All AE costs were calculated by micro-costing of the necessary procedures using Brazilian supplementary health system’s reference costs, according to the 2022 Brazilian Hierarchical Classification of Medical Procedures (CBHPM) table [21]. The mean costs for heart and hepatobiliary diseases were calculated by the cost per event and the number of events. The weighted mean determined the average cost. All costs are presented in Table 3.

**Table 3:** Average cost of adverse events.

System organ class	Adverse event	Cost per event (BRL)	All events (n)	Source
**Cardiac**	Cardiac Arrest	1,120.24	7	CBHPM, 2022 [21]
	Tachycardia	1,120.24	17	
**Average cost (BRL)**			1,120.24	Calculation
**Hepatobiliary**	Hyperbilirubinemia	1,617.53	12	CBHPM, 2022 [21]
	Abnormal hepatic function	249.14	10	
	Jaundice	621.64	5	
	Cholestasis	291.28	5	
**Average cost (BRL)**			827.10	Calculation

BRL: Brazilian real.

The estimated nephrotoxicity treatment cost was included for patients receiving L-anfB. This cost was calculated by hospitalization cost, the incidence of nephrotoxicity among patients treated with L-anfB, and extra hospitalization days needed, generating the value of 5,732.64 BRL [19,22,23]. The cost of dialysis was not included in the model.

#### Patient monitoring—Laboratory analyses

Laboratory monitoring tests were estimated considering the treatment duration and the AEs included in the package leaflet of each drug. Patients receiving treatment for IA and MI were subjected to liver function tests every 15 days if they were taking isavuconazole and once a week if they were taking voriconazole and L-anfB, conforming SELECT and VITAL study. In the budget impact analyses was considering the cost of serum creatinine and urinalysis tests, beyond the cost of liver function test. All costs were calculated from the SHS reference costs, according to the 2022 CBHPM table [21] (Table 4).

**Table 4:** Costs of laboratory tests.

Item	Price (BRL)	Source
Albumin	9,65	CBHPM, 2022 [21]
Total bilirubin and fractions	9,65	
Alkaline phosphatase	17,72	
GammaGT	17,72	
AST	17,72	
ALT	17,72	
Total liver function	90,18	Calculation
Serum creatinine	17,72	CBHPM, 2022 [21]
Urinalysis	20,71	

ALT: alanine transaminase; AST: and aspartate transaminase; BRL: Brazilian real; GammaGT: gamma-glutamyl transferase.

### Incremental cost-utility analysis

The results are based on the incremental cost-utility ratio (ICUR), the cost-utility assessment’s main metric. This ratio is calculated from the assessment’s quality-adjusted life years (QALYs).

Costs and results were discounted with a discount rate of 5% according to the recommendations of the Methodological Guidelines for Studies of Economic Evaluation of Health Technologies, published by the Brazilian Ministry of Health [24].

The analysis results were verified with the willingness-to-pay threshold (WTP) of 40,688.00 BRL per additional QALY gain, corresponding to 1 GDP per capita in Brazil in 2021. Therefore, the evaluated strategy is cost-effective when the ICUR < 40,688.00 BRL/QALY. It is important to note that, currently, there is no established value for a willingness-to-pay threshold in SHS.

### Sensitivity analysis

When conducting an economic study, it is crucial to consider uncertainty quantification in the results and identify the key variables that impact this uncertainty in decision-making.

An univariate sensitivity analysis of the key parameters within the lower and upper limits evaluated the robustness of the base case result (Table 5). The incidence rate of IFI depends on the conditions of the health services provided and may vary according to the region of the country. The IM prevalence rate varied by ±55% to encompass the data found by Bergamasco et al. (2021) in São Paulo [3].

**Table 5:** Lower and upper limits used in sensitivity analysis.

Parameter	Change (%)
Voriconazole–IV price	-25, +25
Percentage requiring second-line treatment	-25, +25
Mortality–Isavuconazole IA	-25, +25
Mortality–Isavuconazole mucormycosis	-25, +25
Mortality–Delayed therapy	-30, +20
Mortality–Untreated	-30, +4
Quality of life estimate	-20, +20
Life expectancy	-25, +25
Treatment durations	-25, +25
Mucormycosis prevalence	-55, +55
Pathogen identification information percentage	-25, +25
Discount rate	-80,+100%

IV: intravenous, IA: invasive aspergillosis

A probabilistic sensitivity analysis was also performed. The model was repeatedly run 1,000 times. Each time, a value was randomly selected for each of the respective different inputs of the probability distribution (Monte Carlo simulations). The average costs and QALYs were calculated with these values, and the results were later summarized.

### Budget impact

The analysis was performed over a time horizon of 5 years from the perspective of the Brazilian SHS from the comparison of costs used for the treatment of patients with IA in the current scenario, in which 75% of patients are treated with voriconazole, and 25% of patients start treatment with L-anfB and switch to oral voriconazole, with the scenario designed with the introduction of isavuconazole for the treatment of IA.

#### Target population

The population eligible for treatment was estimated by the epidemiological method, as shown in Table 6. The base population was the 2022 IBGE projection Brazilian population [25].

**Table 6:** Epidemiological funnel.

General population	Value	Source
National population (n)		IBGE, projection
Population growth per year (%)	0.7	Calculation
Population 18 and over (%)	75	Calculation
Population in private health system	23	ANS, 2022 data
**Invasive aspergillosis**		
Incidence per 100,000	4.47	Giacomazzi, 2016 [26]
IA growth per year (%)	2.7	Bitar, 2014 [12]
Diagnosed population (%)	80	Assumption

IA: invasive aspergilosis

#### Scenarios

Currently, 75% of patients initiate their treatment with voriconazole, while 25% begin with L-anfB and then switch to oral voriconazole. In the projected scenario, isavuconazole is introduced for treating IA patients according to the penetration rate shown in Table 7.

**Table 7:** Scenarios.

**Current scenario**	**Year 1 (%)**	**Year 2 (%)**	**Year 3 (%)**	**Year 4 (%)**	**Year 5 (%)**
Isavuconazole	0	0	0	0	0
Voriconazole	75	75	75	75	75
L-anfB/Voriconazole	25	25	25	25	25
**Projected Scenario**	**Year 1 (%)**	**Year 2 (%)**	**Year 3 (%)**	**Year 4 (%)**	**Year 5 (%)**
Isavuconazole	20	30	40	45	50
Voriconazole	60	53	45	41	38
L-anfB/Voriconazole	20	18	15	14	13

#### Sensitivity analysis

The parameters subject to uncertainty in the budget impact model are the population percentage in the private health system, IA and IM incidence, isavuconazole market share from the first to the fifth year, and the cost of the drugs. These parameters were submitted to variation in univariate sensitivity analysis with an arbitrary interval of ±25%.

## Results

### Base case analysis

Treatment of patients with suspected IA with isavuconazole was associated with an average of 2.61 QALYs. The voriconazole-treated arm was associated with an average of 2.52 QALYs. Therefore, isavuconazole yielded a gain of 0.10 QALYs. From the perspective of the SHS, isavuconazole and voriconazole are associated with a cost of 482,442.00 BRL and 577,616.00 BRL, respectively. Thus, comparing isavuconazole with voriconazole results in a dominant ICUR; which indicates that isavuconazole is a more effective technology that generates resource savings than voriconazole (Table 8).

**Table 8:** Cost-utility results.

	Voriconazole	Isavuconazole
**Base case**		
Total QALYs	2.52	2.61
Incremental QALYs		0.10
Total cost (BRL)	577,616.00	482,442.00
Incremental cost (BRL)		- 95,174.00
ICUR—Incremental cost/QALY		**DOMINANT**
**Alternative Scenario (3-year horizon)**		
Total QALYs	1.58	1.64
Incremental QALYs		0.06
Total cost (BRL)	577,616.00	482,442.00
Incremental cost (BRL)		- 95,174.00
ICUR—Incremental cost/QALY		**DOMINANT**
**Alternative Scenario (10-year horizon)**		
Total QALYs	4.49	4.66
Incremental QALYs		0.17
Total cost (BRL)	577,616.00	482,442.00
Incremental cost (BRL)		- 95,174.00
ICUR—Incremental cost/QALY		**DOMINANT**

BRL: Brazilian real; ICUR: incremental cost-utility ratio; QALY: quality-adjusted life years.

### Sensitivity analysis

**Fig 2:**
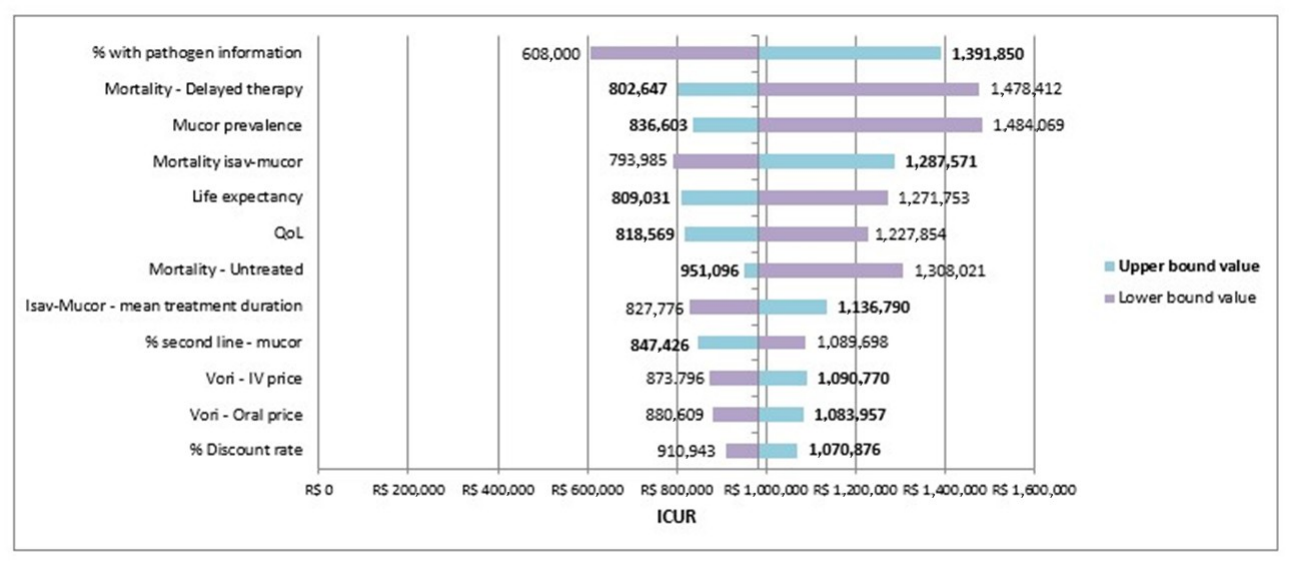
Result of univariate sensitivity analysis of the base case. Mucor: mucormycosis; Isav: isavuconazole; QoL: quality of life; Vori: voriconazole; IV: intravenous; ICUR: incremental cost-utility ratio.

**Fig 3:**
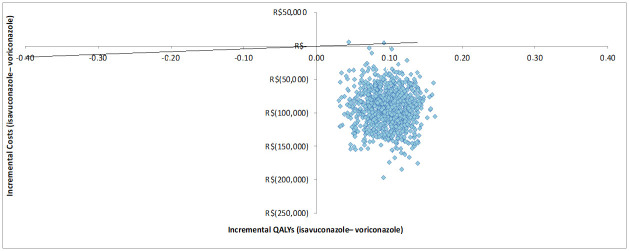
Result of the probabilistic sensitivity analysis of the base case. QALY: quality-adjusted life years.

According to the univariate sensitivity analysis, the ICURs of isavuconazole are dominant versus voriconazole, and the parameters with the most significant impact on the base case analysis were the rate of patients with pathogen identification and mortality due to delay in treatment (Fig 2). According to the willingness-to-pay of 40,688.00 BRL, isavuconazole was a dominant alternative in 99.8% of the Monte Carlo simulations from the SHS perspective (Fig 3).

### Analysis of alternative scenarios with time horizon variation

Alternative scenarios comparing isavuconazole with voriconazole in 3- and 10-year horizons also resulted in dominant ICURs (Table 8). According to the willingness-to-pay of 40,688.00 BRL, isavuconazole was a dominant alternative in 99.7% and 100% of the Monte Carlo simulations, from the SHS perspective, for the horizon of 3 years and 10 years, respectively.

### Budget impact of the isavuconazole inclusion for the treatment of IA patients

#### Target population

According to the epidemiological method, there were an estimated 1,325 eligible patients for IA treatment in the Brazilian SHS in the first year of analysis. This number increased to 1,399 patients in the fifth year (Table 9).

**Table 9:** Target population.

General population	Base year	Year 1	Year 2	Year 3	Year 4	Year 5
National population (n)	214,828,540					
Population growth per year (%)	0.7	214,828,540	216,332,540	217,846,666	219,371,593	220,907,194
Population 18 and over (%)	75	161,121,405	162,249,255	163,385,000	164,528,695	165,680,395
Population in private health system (%)	23	37,057,923	37,317,329	37,578,550	37,841,600	38,106,491
**Invasive aspergillosis**						
Incidence per 100,000	4.47	1,656	1,668	1,680	1,692	1,703
IA growth per year (%)	2.7	1,656	1,713	1,725	1,737	1,749
Diagnosed population (%)	80	1,325	1,370	1,380	1,390	1,399

IA: invasive aspergillosis.

From the number of patients eligible for IA treatment in the SHS, it was possible to estimate, through the market penetration of the drugs used in the treatment, the number of patients who would receive each of the available therapies (Table 10).

**Table 10:** Patients by scenario.

**Current scenario**	**Year 1**	**Year 2**	**Year 3**	**Year 4**	**Year 5**
Isavuconazole	0	0	0	0	0
Voriconazole	994	1028	1035	1042	1050
L-anfB/Voriconazole	331	343	345	347	350
**Projected scenario**	**Year 1**	**Year 2**	**Year 3**	**Year 4**	**Year 5**
Isavuconazole	265	411	552	625	700
Voriconazole	795	720	621	573	525
L-anfB/Voriconazole	265	240	207	191	175

#### Budget impact

After analyzing the market penetration of isavuconazole, it was discovered that incorporating it through the SHS results in significant savings compared to voriconazole. In the first year, 20% market penetration of isavuconazole can lead to savings of around 20.5 million BRL. By the fifth year, with a market penetration of 50%, savings are estimated to reach around 54 million BRL (Table 11).

**Table 11:** Budget impact result—Base case.

	Year 1	Year 2	Year 3	Year 4	Year 5	Total
**Net Budget Impact (BRL)**	- 20.487.179,20	- 31.781.423,06	- 42.671.857,36	- 48.341.880,41	- 54.089.192,86	- 197.371.532,90
**Scenario without isavuconazole**						
Voriconazole (BRL)	194.654.388,97	201.309.427,88	202.718.593,87	204.137.624,03	205.566.587,40	1.008.386.622,14
AmBisome > Posaconazole (BRL)	-	-	-	-	-	-
AmBisome > Voriconazole (BRL)	103.539.854,27	107.079.778,34	107.829.336,79	108.584.142,15	109.344.231,14	536.377.342,70
**Total (BRL)**	**298.194.243,24**	**308.389.206,22**	**310.547.930,66**	**312.721.766,18**	**314.910.818,54**	**1.544.763.964,84**
**Scenario with isavuconazole**						
Isavuconazole (BRL)	39.151.669,44	60.735.338,80	81.547.314,90	92.382.914,37	103.366.216,41	377.183.453,92
Voriconazole (BRL)	155.723.511,18	140.916.599,51	121.631.156,32	112.275.693,22	102.783.293,70	633.330.253,92
AmBisome > Posaconazole (BRL)	-	-	-	-	-	-
AmBisome > Voriconazole (BRL)	82.831.883,41	74.955.844,84	64.697.602,08	59.721.278,18	54.672.115,57	336.878.724,08
**Total (BRL)**	**277.707.064,03**	**276.607.783,16**	**267.876.073,30**	**264.379.885,77**	**260.821.625,68**	**1.347.392.431,93**

BRL: Brazilian real.

#### Sensitivity analysis

**Fig 4:**
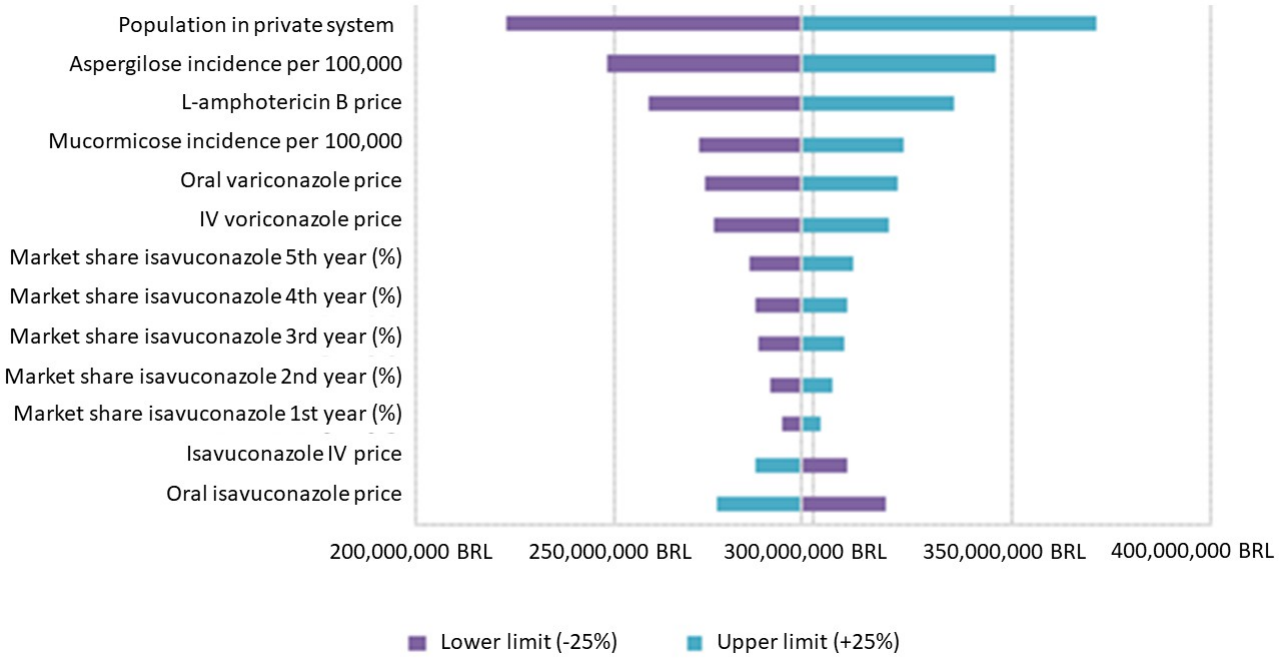
Univariate sensitivity analysis result. IV: intravenous.

The results of the univariate sensitivity analysis were expressed in a Tornado diagram (Fig 4; Table 12) that represents the variation of the total incremental impact in 5 years resulting from the variation of the values of each parameter according to the proposed intervals.

**Table 12:** Univariate sensitivity analysis—Parameters and results.

Parameter	Base scenario	Minimum (-25%)	Maximum (+25%)	Total incremental impact in 5 years Lower limit (-25%)—BRL	Total incremental impact in 5 years Upper limit (+25%)—BRL
% Population in private system	23	17.25	28.75	- 148,028,649.68	- 246,714,416.13
Aspergilose incidence per 100,000	4.47	3.3525	5.5875	- 148,028,649.68	-246,714,416.13
L-amphotericin B price—BRL	24,010.96	18,008.22	30,013.70	- 170,607,416.95	- 224,135,648.86
Oral voriconazole price—BRL	6,951.54	5,213.65	8,689.42	- 173,009,244.59	- 221,733,841.22
IV voriconazole price—BRL	1,688.21	1,266.16	2,110.26	- 175,507,132.30	- 219,235,881.70
Market share isavuconazole 5th year (%)	50	37.5	62.5	- 183,849,234.69	- 210,893,813,12
Market share isavuconazole 4th year (%)	45	33.75	56.25	- 185,286,062.80	- 209,457,003.01
Market share isavuconazole 3rd year (%)	40	30	50	- 186,703,568.56	- 208,039,497.25
Market share isavuconazole 2nd year (%)	30	22.5	37.5	- 189,426,177.14	- 205,316,888.67
Market share isavuconazole 1st year (%)	20	15	25	- 192,249,738.10	- 202,493,327.71
Mucormicose incidence per 100,000	0.2	0.15	0.25	- 197,371,532.90	- 197,371,532.90
Isavuconazole IV price–BRL	1,734.77	1,301.08	2,168.46	- 207,420,917.69	- 187,322,148.12
Oral isavuconazole price—BRL	4,571.83	3,428.87	5,714.79	- 214,850,511.20	- 179,892,544.61

BRL: Brazilian real; IV: intravenous.

The parameters of greatest influence in the analysis were the percentage of the population in the SHS, the IA and L-amphotericin B price, as well as the Oral and IV voriconazole price.

## Discussion

From the SHS perspective, the economic analyses of isavuconazole comparing voriconazole, demonstrated that isavuconazole is dominant because it promoted greater utility gain for patients at a lower cost. The budget impact analysis result supports the cost-utility analysis by demonstrating that the introduction of isavuconazole to patients with suspected IA will promote savings to the SHS. This outcome can be attributed to the benefits of isavuconazole compared to voriconazole. These benefits include fewer adverse events, better tolerance, reduced need for patient monitoring tests, and shorter hospital stays.

Previous studies have compared the cost-effectiveness of isavuconazole and voriconazole in the Swedish, Spanish, and Canadian healthcare systems. The results show that isavuconazole is a cost-effective option for treating patients suspected of having IA in all three settings [5–7].

The cost-utility analyses of isavuconazole versus voriconazole with scenario variations were published in the abstract at ISPOR Europe, 2022 [27]. This study aimed to verify the impact of the acquisition cost of reference or generic voriconazole. In this publication, the analyses showed that isavuconazole remains dominant regardless of whether the comparator is a voriconazole reference or generic. In another scenario simulation, a discount of 60% was applied to the acquisition costs of the reference and generic voriconazole to simulate the current market practice of this product in Brazil. Even with the application of this discount, the results remained dominant for isavuconazole.

This model is an open tool adaptable to other values. Among the model limitations is the discount rate of 5%. Although this rate is reviewed by the Methodological Guidelines for Studies of Economic Assessment of Health Technologies [24], inflation in Brazil can reach around 10%, and healthcare inflation can present higher rates. Regarding the daily hospitalization cost, we used the average data from the self-management operators, which does not reflect the costs of all Brazilian healthcare operators. This value correction by IGP-M, FGV [20] may also not reflect the healthcare inflation. Costs related to imaging and dialysis were not considered in the model.

The model also assumed the use of second-line L-anfB treatment. However, many healthcare services in Brazil still use amphotericin B deoxycholate, which, despite the lower acquisition cost, is associated with high rates of AEs and lower efficacy when compared to L-anfB [28,29].

It is worth mentioning that the SHS cost data available in the literature are mean values, but the costs practiced by health operators are higher. We can conclude that the presented analyses are conservative based on the potential difference. Using isavuconazole for patients with suspected IA in the SHS would likely result in even greater savings than what was shown in the budget impact analysis.

## Conclusion

The study demonstrated that isavuconazole incorporation by the SHS is a dominant strategy; that is, it promotes greater benefit at a lower total treatment cost to patients with suspected IA compared to voriconazole, generating savings to the system.

## Supporting information

S1 File (ZIP)
